# Syndrome of Inappropriate Antidiuretic Hormone Secretion and Ibuprofen, a Rare Association to Be Considered: Role of Tolvaptan

**DOI:** 10.1155/2013/818259

**Published:** 2013-06-02

**Authors:** Nathan Artom, Silvia Oddo, Aldo Pende, Luciano Ottonello, Massimo Giusti, Franco Dallegri

**Affiliations:** ^1^Clinic of Internal Medicine 1, Department of Internal Medicine, University of Genoa School of Medicine, Viale Benedetto XV, 6, 16132 Genoa, Italy; ^2^Division of Endocrinology, Department of Endocrinological and Metabolic Sciences, University of Genoa School of Medicine, Genoa, Italy

## Abstract

The association between the syndrome of inappropriate antidiuretic hormone secretion (SIADH) and the use of nonsteroidal anti-inflammatory drugs (NSAIDs) is rare and has never been treated with an arginine vasopressin receptor antagonist. We report a unique case of SIADH associated with ibuprofen use and successfully treated with tolvaptan. A 76-year-old man came to our observation because of lumbar pain and epigastric discomfort. He was taking ibuprofen orally 400 mg bid as an analgesic treatment. Laboratory tests showed low levels of sodium (116 mmol/L) and chloride; a diagnosis of SIADH was formulated and ibuprofen was stopped immediately. Imaging tests allowed to rule out the presence of malignancies or cerebral and lung diseases. Slightly hypertonic saline infusion was administered for 3 days without significant sodium improvement; therefore, tolvaptan was started at the initial dose of 7.5 mg daily, doubled after 5 days. After 8 days of treatment the patient showed progressive increase of sodium levels up to normal values. In the following weeks tolvaptan was prescribed at progressively titrated dosage to full suspension; afterwards the sodium levels remained normal without any type of treatment.

## 1. Introduction

Hyponatremia is the most frequent electrolyte abnormality in hospitalized patients and is associated with a greatly increased morbidity and mortality; in this context the syndrome of inappropriate antidiuretic hormone secretion (SIADH) is the most frequent cause of hyponatremia [1]. 

The diagnostic criteria for the diagnosis of SIADH are hypoosmolality (plasma osmolality <280 mOsm/kg, or plasma sodium concentration <134 mmol/L); inappropriate urinary osmolality concentration (Uosm >100 mOsm/kg) for hyponatremia; elevated urinary sodium (>40 mmol/L), with normal dietary salt and water intake; patient's normovolemia; exclusion of hypothyroidism, diuretic treatment, and glucocorticoid deficiency [2]. The most common causes of SIADH are malignancies, pulmonary disorders, central nervous system disorders, and medications. 

Usual therapeutic options in hyponatremic patients with SIADH consist of fluid restriction (less than 800–1200 mL daily), hypertonic saline solution, furosemide with oral salt supplementation, oral urea, and demeclocycline. The choice of the therapeutic strategy depends on the symptoms of the patient, the acute or chronic onset, and the severity of hyponatremia [2]. Tolvaptan is a new drug that targets the mechanism of the disorder: it is an orally active, selective, nonpeptide antagonist that blocks arginine vasopressin (AVP) binding to V_2_ receptors of the distal nephron and consequently induces the excretion of electrolyte-free water without changes in the level of sodium excretion [3]. 

The two most important studies reporting tolvaptan treatment of normovolemic or hypervolemic hyponatremia are the SALT and the EVEREST trials [3, 4]. In patients with normovolemic or hypervolemic hyponatremia, the SALT study showed that tolvaptan treatment significantly increased the serum sodium levels on days 4 and 30 of the study [3]. In patients hospitalized for heart failure, the EVEREST study showed that tolvaptan treatment had no effects on long-term mortality or heart failure-related morbidity [4].

Our report describes a case of severe SIADH due to a nonsteroidal anti-inflammatory drug (NSAID), the first reported in literature treated with an oral AVP-receptor antagonist.

## 2. Case Report

A 76-year-old Caucasian man came to our observation because of lumbar pain and epigastric discomfort. He was taking ibuprofen orally 400 mg *bid *as an analgesic treatment for 1 month. About his clinical history, 1 year before the admission, he had suffered from acute coronary syndrome during an episode of atrial tachyfibrillation treated with percutaneous transluminal coronary angioplasty and stenting, and subsequently a biologic prosthetic mitral valve had been implanted due to severe mitral insufficiency. He was on regular treatment with warfarin, atorvastatin, and bisoprolol tablets. At the admission to our ward we stopped ibuprofen. He demonstrated a state of mild confusion and irritability. Laboratory tests showed sodium 116 mmol/L and chloride 84 mmol/L; hepatic and renal tests, as well as the other routine blood tests, were normal, with uric acid 2.5 mg/dL and urea 37 mg/dL. Duration of hyponatremia was unknown. The patient appeared clinically normovolemic (blood pressure 126/76 mmHg without orthostatic hypotension, heart rate 86 bpm) and showed no signs of heart or renal failure. We performed thyroid function tests and measurement of urinary cortisol excretion that resulted normal. After the evidence of low plasma osmolality (252 mOsm/L), high Uosm (561 mOsm/L), and high urinary sodium (160 mmol/L), a diagnosis of SIADH was formulated. To determine the etiopathogenesis of the syndrome, we performed brain MR, total body CT scan, radionuclide bone scan, serologic tests for *Chlamydia*, *Legionella*, and *Mycoplasma*: all tests resulted normal.

**Figure 1:**
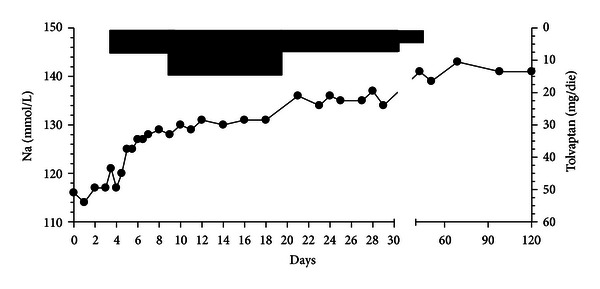
Time course of serum sodium levels during tolvaptan treatment.

We started infusion of a slightly hypertonic saline solution (1.2% at a rate of 60 mL per h) for 3 days without significant sodium changes; since the patient was taking warfarin and therefore we could not prepare immediately a central iv line to allow the infusion of a more hypertonic solution (3% saline), we decided to treat the patient with tolvaptan, an oral AVP V_2_ receptor antagonist, initially at the dosage of 7.5 mg daily, checking plasmatic sodium level every 6 hours, and after 5 days at the dosage of 15 mg daily. The patient was encouraged to drink water if he felt thirsty. The changes of sodium level associated with the treatment are presented in Figure 1. After 8 days of treatment the patient showed progressive increase of sodium levels up to normal values and fully recovered clinically. In the following weeks tolvaptan was prescribed at progressively titrated dosage to full suspension (at day 40); afterwards the sodium levels remained normal without any type of treatment.

Our diagnostic conclusion was drug-induced SIADH, due to the use of ibuprofen, with a complete recovery of normal sodium levels induced by the treatment with a V_2_ receptor antagonist.

## 3. Discussion

SIADH is the most frequent cause of euvolemic hyponatremia, a condition characterized by increased morbidity and mortality [1]. Despite being described more than 50 years ago, the management of SIADH is still controversial [5]; in addition in recent years a new therapeutic option, the AVP receptor antagonists, also known as aquaretics or vaptans, was introduced [6].

Drugs are a common cause of SIADH and in particular the various groups of antidepressants are frequently involved in this important electrolyte disturbance [7]. Our patient showed a severe hyponatremia after a prolonged use of ibuprofen, a less common inducer of SIADH. NSAIDs exert their pharmacological effects through the inhibition of the synthesis of prostaglandins [8]: at the kidney level prostaglandins are involved in the modulation of both glomerular filtration rate and tubular sodium reabsorption. In healthy hydrated individuals renal prostaglandins do not exert a major role in sodium and water homeostasis but in conditions such as chronic heart failure, cirrhosis, chronic nephropathy, and hypovolemic states, NSAIDs decrease renal blood flow and glomerular filtration rate significantly [9]. In addition these drugs can reduce water excretion by specifically potentiating the renal effects of AVP, although at the central level prostaglandin inhibition may suppress AVP secretion [7]. Our patient did not show any evidence of either hemodynamic impairment or decrease of renal function, apparently presenting a condition of pure inappropriate AVP secretion.

Traditional approaches to hyponatremia management in SIADH patients are limited by the challenge of compliance, barriers in the practicality of fluid restriction, and risk of serious neurological adverse effects or worsening congestion in patients with hypervolemia, both related to saline infusion. Lithium and demeclocycline have also been proposed to correct hyponatremia, but their use is limited by adverse effects, particularly nephrotoxicity. In this context the antagonists of AVP receptors are a new opportunity and a drug of this class, tolvaptan, was recently approved by EMEA for the oral treatment of hyponatremia due to SIADH in the European Union [10, 11]. AVP binds to 3 different receptors: V_1a_ receptors are present on vascular smooth muscle cells, V_1b_ are detected in the anterior pituitary and pancreas modulating ACTH and insulin release, and V_2_ receptors are detected in the principal cells of the renal collecting duct [12]. Tolvaptan is a specific oral antagonist of V_2_ receptors inducing an increase in solute-free water excretion, with minimal-to-no effect on electrolyte excretion, termed the “aquaresis effect” [12]: it has been evaluated in patients with both euvolemic and hypervolemic hyponatremia [10].

Our patient was not treated with fluid restriction because sodium levels were too low (recommended water consumption should have been close to 0 mL) [6]: we started with a slightly hypertonic saline solution which did not induce any significant change in hyponatremia and, after 3 days, we decided to begin tolvaptan. At the dosage of 7.5 mg daily tolvaptan induced an increase of 9 mmol/L in serum sodium levels after 24 hours and 11 mmol/L after 48 hours; in the subsequent days, with an increased dosage for tolvaptan of 15 mg daily, the sodium improvement was less marked with the achievement of normal values (>135 mmol/L) after 13 days of treatment. The drug was very well tolerated without any evidence of osmotic demyelination syndrome. 

The dose of tolvaptan was quite low compared to the doses used in both the SALT trials and the EVEREST trial [4]: we decided to start with a daily dose of 7.5 mg of tolvaptan, only doubling it after a few days. We obtained a very good response after the first administration of tolvaptan (plus the suspension of ibuprofen) and therefore we did not increase the drug to the suggested daily doses of 30–60 mg. However, after the significant initial increase in sodium levels, we experienced some difficulties in reaching normal values (7 days to arrive at 130 mmol/L, 13 days to arrive at 135 mmol/L): therefore we maintained the drug for a longer period with respect to the SALT population [10]. Recently Meyer et al. administered tolvaptan in a severe hyponatremia (108 mmol/L) induced by the antidepressant venlafaxine, obtaining a more rapid, and possibly dangerous, rise in sodium levels (to 131 mmol/L in 2 days) [20]. A possible explanation of these discrepancies could be related to the complex effects of a NSAID to renal functions, not only in terms of potentiation of the AVP effects but also in terms of independent effects on glomerular filtration rate and tubular sodium reabsorption [21]. 

In all cases of SIADH, such as those evaluated in the SALT-1 and SALT-2 trials, tolvaptan was superior to placebo in increasing serum sodium levels, usually with a good tolerability and safety; additional clinical benefits of tolvaptan were represented by the lack of need for fluid restriction and by the improvement of physical and mental symptoms [10]. Possible decreases in morbidity and mortality remain to be evaluated by future clinical trials. Recently another evaluation of the data of the SALT-1 and SALT-2 trials allowed Dasta et al. to demonstrate that tolvaptan use is associated with a shorter length of hospital stay than placebo among patients with SIADH; considering the drug cost for inpatients therapy, tolvaptan was associated with a significant estimated mean hospital cost reduction in the United States [22].

In the available literature the correlation between NSAIDs and SIADH is anecdotal [13–19].  Table 1 shows a summary of the case reports. Most cases of NSAID-induced SIADH were caused by ibuprofen: they were usually characterized by severe hyponatremia (sodium <120 mmol/L) and most of the times treated with fluid restriction. No reported case was probably treated with hypertonic saline. Some of those cases are only associated with hyponatremia, and it is not reported whether hyponatremia is in the context of a SIADH diagnosis. Moreover cases of NSAID-induced SIADH, treated with tolvaptan, are not reported in the literature. 

Our contribution shows the importance of investigating the use of the NSAIDs in the pharmacological anamnesis of SIADH patients and how tolvaptan can be a good strategy in NSAID-induced SIADH. 

## Figures and Tables

**Table 1:** Published cases of NSAID-induced SIADH.

Authors	Reference	No. of pts.	Initial sodium levels (mmol/L)	Therapy	Drug involved
Temple et al.	[13]	2	131–134	Mannitol/fluid restriction	Salicylate
Blum and Aviram	[14]	1	115	Fluid restriction	Ibuprofen
Dunn et al.	[15]	2	118 118	Fluid restriction Fluid restriction	Ibuprofen Diclofenac
Petersson et al.	[16]	2	119 120	Hypertonic saline Isotonic saline/fluid restriction	Piroxicam Diclofenac
Cheung et al.	[17]	1	116	Fluid restriction	Nabumetone/diclofenac
Rault	[18]	1	125	Isotonic saline	Ibuprofen
Shimada et al.	[19]	1 (twice)	118 104	Fluid restriction	Metamizole Propyphenazone
